# Characterization of fragment sizes, copy number aberrations and 4‐mer end motifs in cell‐free DNA of hepatocellular carcinoma for enhanced liquid biopsy‐based cancer detection

**DOI:** 10.1002/1878-0261.13041

**Published:** 2021-07-16

**Authors:** Chao Jin, Xiaonan Liu, Wenyuan Zheng, Liping Su, Yang Liu, Xu Guo, Xiaoming Gu, Hongping Li, Bo Xu, Gang Wang, Jiyan Yu, Qiong Zhang, Dengke Bao, Shaogui Wan, Fei Xu, Xiaohuan Lai, Jiayun Liu, Jinliang Xing

**Affiliations:** ^1^ Department of General Surgery Tangdu Hospital Fourth Military Medical University Xi’an China; ^2^ Ambulatory Surgery Center of Xijing Hospital Fourth Military Medical University Xi’an China; ^3^ Research and Development Division Oriomics Biotech Hangzhou China; ^4^ State Key Laboratory of Cancer Biology Department of Physiology and Pathophysiology Fourth Military Medical University Xi’an China; ^5^ Laboratory of Cancer Biomarkers and Liquid Biopsy School of Pharmacy Henan University Kaifeng China; ^6^ Center for Molecular Pathology First Affiliated Hospital Gannan Medical University Ganzhou China; ^7^ Department of Hepatology The Fifth People’s Hospital of Ganzhou China; ^8^ Department of Clinical Laboratory Xijing Hospital Fourth Military Medical University Xi’an China

**Keywords:** circulating cell‐free DNA, copy number variation, end motifs, fragment sizes, hepatocellular carcinoma, tumor fraction

## Abstract

Circulating cell‐free DNA (cfDNA) fragmentomics, which encompasses the measurement of cfDNA length and short nucleotide motifs at the ends of cfDNA molecules, is an emerging field for cancer diagnosis. The utilization of cfDNA fragmentomics for the diagnosis of patients with hepatocellular carcinoma (HCC) caused by hepatitis B virus (HBV) is currently limited. In this study, we utilized whole‐genome sequencing data of cfDNA in samples from patients with HCC (*n* = 197) and HBV (*n* = 187) to analyze the association of fragment size selection (< 150 bp) with tumor fraction (TF), copy number variation (CNV) alterations and the change in the proportion of 4‐mer end motifs in HCC and HBV samples. Our analyses identified five typical CNV markers (i.e. loss in chr1p, chr4q and chr8p, and gain in chr1q and chr8q) in cfDNA with a cumulatively positive rate of ˜ 95% in HCC samples. Size selection (< 150 bp) significantly enhanced TF and CNV signals in HCC samples. Additionally, three 4‐mer end motifs (CCCA, CCTG and CCAG) were identified as preferred end motifs in HCC samples. We identified 139 end motifs significantly associated with fragment size that showed similar patterns of associations between patients with HCC and HBV, suggesting that end motifs might be inherently coupled with fragment size by a ubiquitous mechanism. Here we conclude that CNV markers, fragment size selection and end‐motif pattern in cfDNA have potential for effective detection of patients with HCC.

## Graphical abstract

Circulating cell‐free DNA (cfDNA) fragmentomics, encompassing the measurement of cfDNA length and short nucleotide motifs at cfDNA ends, is an emerging field in cancer diagnostics. In this study, we utilized whole‐genome sequencing data of cfDNA in patients with hepatocellular carcinoma (HCC). We observed that representative abnormal copy number variation (CNV) alterations and shorter fragment size (< 150 bp) selection enhanced the accuracy of CNV detection and improved the clinical utility of ctDNA in HCC. We also discovered a strong correlation of end motif type to fragment size and revealed similar fragment size characteristics of 4‐mer end motifs between patients with HCC and hepatitis B virus.

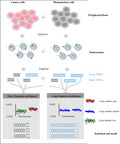

**Abbreviations**
cfDNA: circulating cell‐free DNA
ctDNA: circulating tumor DNA
CNV: copy number variation
HBV: hepatitis B virus
HCC: hepatocellular carcinoma
TF: tumor fraction
WGS: whole‐genome sequencing

## 1 Introduction

Hepatocellular carcinoma (HCC) is the sixth most common cancer and fourth leading cause of cancer death, with 841 000 new cases and 781 000 death worldwide in 2018 [1]. As one of the main risk factors of HCC, hepatitis B virus (HBV) is responsible for 35% of deaths from HCC [2]. HBV may contribute to HCC through integrating HBV DNA into host genome to mediate genetic abnormality and influence the expression of HCC‐related genes [3]. HBV infection is the leading cause of HCC in Asian and African countries, especially in China [2, 4]. Early diagnosis and effective control are important to reduce the social burden induced by HBV‐infected HCC. To date, although tumor biopsy remains the gold standard for HCC diagnosis, there is controversy concerning reliability in small nodule cases and adverse consequences such as bleeding and intraprocedural hematogenous dissemination [5]. For HBV‐infected HCC with poor liver function, it is urgent to search for new potential biomarkers based on noninvasive methods.

Circulating cell‐free DNA (cfDNA) are short fragments detectable in blood or other body fluids shed from different cell types. Recent advances in combined cfDNA analysis and the whole‐genome sequencing (WGS) method have contributed to the development of noninvasive liquid biopsy for cancer diagnosis as a surrogate for standard tumor biopsies [6]. So far, the characterization of cfDNA in HCC patients has been well reported and has laid the foundation for HCC diagnosis using genomic alteration and DNA methylation‐derived markers such as single‐nucleotide variants and copy number variations (CNV) [7, 8, 9]. However, the biomarkers derived for the diagnosis of HCC patients with HBV infection are still limited. Recently, cfDNA fragmentomics, also called properties or patterns of cfDNA fragmentation, which encompasses fragment sizes, end points and nucleosome footprints, is an emerging field for cancer diagnosis [10, 11, 12]. Cristiano *et al*. applied a machine‐learning model based on incorporated fragmentation features to improve sensitivity and specificity for cancer detection [13]. DNA fragment selection of 90–150 bp was reported to improve the detection of mutated DNA fraction of ovarian cancer up to 11‐fold [14]. However, the cfDNA fragment patterns in cancers, especially in HBV‐HCC, are still poorly understood. Therefore, a comprehensive understanding of the fragmentation features, mechanisms and patterns of cfDNA is important for discovering promising cancer biomarkers in HCC.

Jiang *et al*. [15] recently exploited the DNA end‐motif profiles of HCC samples using massively parallel sequencing and reported a significant increase in the diversity of DNA end motifs in HCC samples. They also reported that HCC subjects had some preferential patterns of 4‐mer end motifs such as CCCA, compared with controls. However, in their study, they focused on HCC patients alone, not HCC patients with HBV infection. Additionally, as both fragment size and motifs are associated with ctDNA, it remains unclear whether these are independent biomarkers to enhance cancer detection. As fragment sizes and end motifs are likely to be involved in DNA cleavage, investigation of fragment sizes and end motifs is of critical importance in further dissecting the mechanisms of cfDNA patterns.

Thus, in this study, we analyzed WGS data (˜ 5× depth coverage on average) of cfDNA from 197 HCC samples with HBV infection and 187 non‐cancer HBV samples [16]. We aimed to screen and identify representative CNV markers for distinguishing the HBV‐HCC samples from controls with HBV infection, and to further select appropriate fragment sizes to improve the tumor fraction (TF) of circulating tumor DNA (ctDNA) in HCC samples. We also attempted to explore the end‐motif patterns as a biomarker for HCC detection and, in particular, evaluate the associations of fragment sizes and 4‐mer end motifs in HCC and HBV samples.

## 2 Materials and methods

### 2.1 Sample characteristics

The WGS data of 197 HBV‐related HCC samples and 187 non‐HCC HBV samples from our previous investigation [16] were utilized in this work. The characteristics of samples were shown in Table 1 of the previous article [16]. The study was approved by the local Ethics Committees of the involved hospitals, and all the subjects provided written informed consent. All study methodologies conformed to the Declaration of Helsinki.

**Table 1:** Detectability of CNA in plasma of HCC patients and HBV carriers. Chr, chromosome.

Subject category	chr1p	chr1q	chr4q	chr8p	chr8q	Any of chr1p/1q/4q/8p/8q
HCC (*n* = 63)	26/63 (41.3%)	50/63 (79.4%)	44/63 (69.8%)	31/63 (49.2%)	44/63 (69.8%)	59/63 (93.7%)
HCC‐TF high (*n* = 19)	11/19 (57.9%)	13/19 (68.4%)	15/19 (78.9%)	17/19 (89.5%)	12/19 (63.2%)	18/19 (94.7%)
HBV (*n* = 187)	0/187 (0.0%)	2/187 (1.07%)	0/187 (0.0%)	0/187 (0.0%)	2/187 (1.07%)	2/187 (1.07%)

### 2.2 Cell‐free DNA isolation, library construction and WGS sequencing

Plasma cfDNA from fresh whole blood was extracted using QIAamp Circulating Nucleic Acid Kit (Qiagen, Hilden, Germany) following the manufacturer’s protocols. The cfDNA concentration and quality were assessed using Qubit 3.0 (Thermo Fisher Scientific, Waltham, MA, USA) and Bioanalyzer 2100 (Agilent Technologies, Palo Alto, CA, USA), respectively. The cfDNA libraries for WGS sequencing were prepared using about 20 ng cfDNA as previously described [16]. Briefly, cfDNA was processed by end repairing, dA tailing and ligation to loop adapter, followed by size selection using AMPure XP beads (Beckman Coulter, High Wycombe, UK). The adaptor‐ligated DNA fragments were amplified by a 14‐cycle PCR using two Illumina p5 and p7 primers. Subsequently, libraries were quantified with the KAPA Library Quantification kit (KAPA Biosystems, Wilmington, MA, USA) and library fragment size was determined by Bioanalyzer 2100. WGS sequencing was then conducted on the Illumina HiSeq X10 platform using a paired‐end 150‐bp protocol.

### 2.3 WGS data processing

Raw paired‐end reads were pre‐processed using fastp (version 0.20.0, https://github.com/OpenGene/fastp) to remove adaptor, low‐quality bases and consensus bases. Quality control of reads was evaluated using QPLOT [17]. The trimmed reads were then aligned to the reference human genome hg19 using BWA (version 0.7.17, https://sourceforge.net/projects/bio‐bwa/files/) [18] and GATK4 markduplicates (version 4.1.2.0, The Apache Software Foundation, Wakefield, MA, USA) was used for masking of BAM files by duplicate reads.

### 2.4 CNV and TF analyses

To estimate the CNV and TF of HCC samples, ichorcna (https://github.com/broadinstitute/ichorCNA/, Broad Institute of MIT, Cambridge, MA, USA) was used to analyze tumor fractions in cfDNA from WGS samples [19]. Briefly, the genome was divided into non‐overlapping 1‐Mb bins, and reads with mapping quality of < 20 were filtered. The mapped reads within each bin were calculated using hmmcopy software in r package (http://bioconductor.org/packages/2.11/bioc/html/HMMcopy.html). After conducting GC content and mappability bias correction, CNA prediction and TF estimation in cfDNA were performed using ichorcna. Segmentation of CNV profiling data was performed using ichorcna, in which an HETD CNV region is defined as loss and a GAIN or AMP CNV region is defined as gain. The maps of genome‐scale overview of cfDNA CNV were drawn using matplotlib (version 3.1.1, https://matplotlib.org/stable/citing.html) package.

### 2.5 cfDNA fragment size analysis

The insert size of an assigned read pair from BAM file was considered the fragment size of a cfDNA fragment. Fragment size distributions of cfDNA were drawn using the matplotlib (version 3.1.1) package. Mean proportions of cfDNA fragments < 150 bp in all fragment sizes from 20 HCC samples with TF > 0.2, 43 HCC samples with 0 < TF ≤ 0.2, and 187 HBV samples were compared using one‐tail *t*‐tests and the results represented by box plots were drawn using the matplotlib package. The effects of selecting fragments of ≤ 150 bp on TF and CNV signal changes in HCC samples were further evaluated. First, read pairs with insert sizes of ≤ 150 bp were extracted to rebuild a new BAM file. Then, the TF after ≤ 150 bp size selection was recalculated through ichorcna software and linear least‐squares regression was conducted using scipy.stats.linregress function in python (https://docs.scipy.org/doc/scipy/reference/generated/scipy.stats.linregress.html) to investigate the correlation between TF values before and after size selection.

### 2.6 cfDNA 4‐mer end‐motif analysis

The cfDNA end motifs were defined using the first 4‐nucleotide sequence of each 5′ end of aligned reads as previously described [15]; only properly paired reads with mapping quality > 20 were used for downstream analyses. There were 256 possible 4‐mer motifs, of which the frequencies of six representative motifs (CCCA, CCTG, CCAG, TAAA, AAAA and TTTT) at the end of sequenced fragments in HCC high‐TF (TF > 0.2), HCC low‐TF (0 < TF ≤ 0.2) and HBV samples were calculated and compared using one‐tail *t*‐tests. The results shown by box plots were drawn by scipy software (http://www.scipy.org). The fragment size distributions of reads with these six representative end motifs were analyzed using matplotlib. Next, changes in the proportion of 256 4‐mer end motifs before and after selecting fragments < 150 bp in 197 HCC samples and 187 HBV samples were calculated. The associations of the proportions of 256 4‐mer end motifs before and after size selection were investigated by linear least‐squares regression. Hierarchical clustering was conducted to analyze the associations of fragment sizes and 70 end motifs using clustermap function in seaborn software (version 0.9.0, https://github.com/mwaskom/seaborn) based on matplotlib; only end motifs with proportions > 0.005 were used.

## 3 Results

### 3.1 CNV alterations of cfDNA in HCC samples based on WGS

**Fig. 1:**
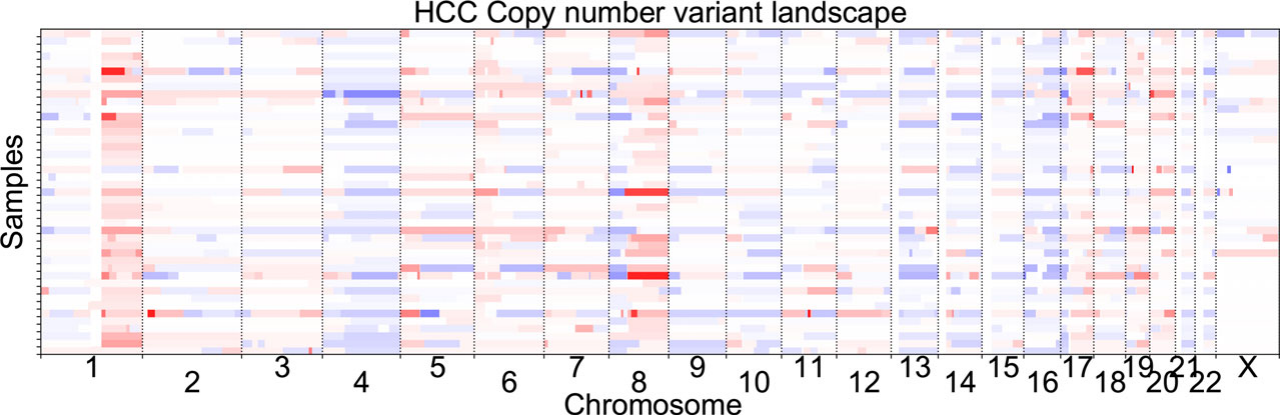
Genome‐scale overview of cfDNA CNV in 63 HCC samples with HBV infection detected by WGS. *X*‐axis represents 1–22 X‐chromosomes and each line in *Y*‐axis represents one HCC sample. CNV amplifications are presented in red and CNV deletions in blue. The darker the color is, the greater the amplitudes/deletions are.

The cfDNA profiles of 63 HCC samples with estimated TF > 0 and 187 HBV samples were analyzed. The CNV of chr1, chr4 and chr8 were commonly affected in HCC, including loss in chr1p, chr4q and chr8p, and gain in chr1q and chr8q (Fig. 1). Our data revealed that 59 of 63 (93.7%) HCC samples exhibited at least one of the above five CNV, and 18 of 19 (94.7%) HCC samples with high‐TF (TF > 0.2) had at least one of these CNV alterations. For HBV samples, none of 187 cases presented the above CNV alterations except for two cases: one (HBV‐275) was detected with chr1q and chr8q amplifications (chr1q: *P* = 3.02e−10; chr8q: *P* = 4.37e‐3; one‐tail *t*‐test comparing the q‐arm and the p‐arm), and another (HBV‐116) was detected with the same CNV aberrations (chr1q: *P* = 7.59e−4; chr8q: *P* = 8.31e−4; Table 1). Unfortunately, both of these two subjects were subsequently diagnosed with HCC during follow‐up, with HBV‐275 being diagnosed at the 3‐month follow‐up and HBV‐116 at the 6‐month follow‐up. The results suggested that the HCC samples had high positive rates of these five typical CNV markers across patient samples, providing valuable biomarkers for distinguishing HCC samples from HBV controls and for early detection of undiagnosed HBV patients 6 months before the clinical diagnosis.

### 3.2 Utilization of cfDNA fragment size to enhance the detection of CNV markers

**Fig. 2:**
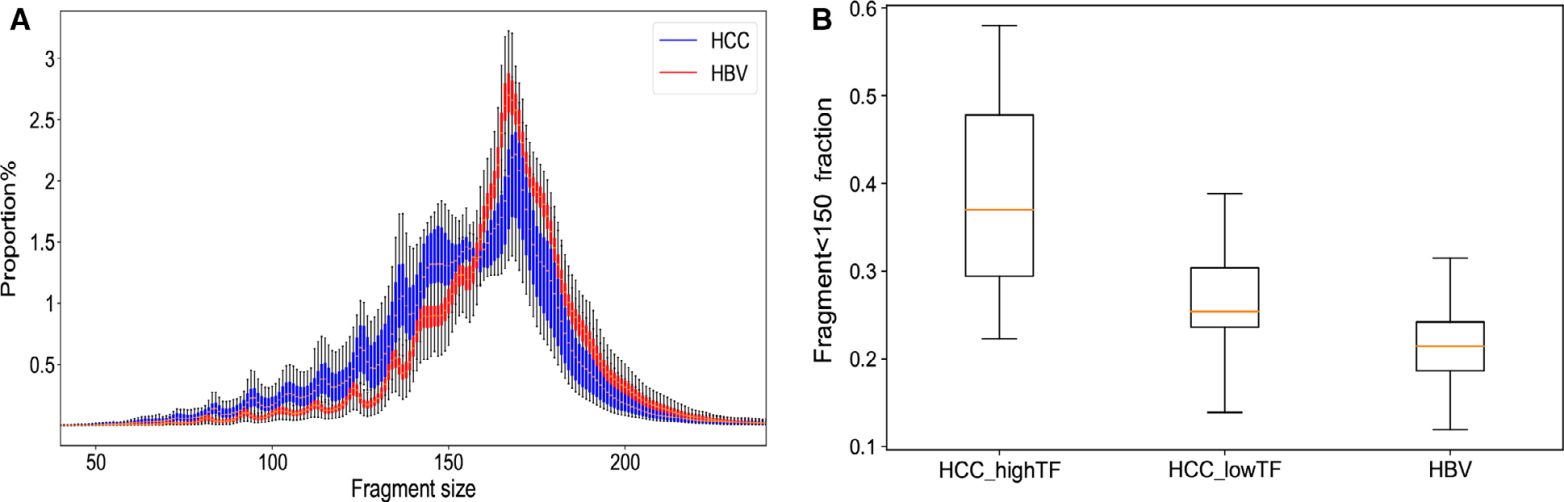
A distinct difference of fragment lengths in HCC and HBV samples. (A) Fragment size density distribution of DNA fragments of 20 HCC samples with TF > 0.2 and 187 HBV samples. *X*‐axis represents the length of fragments and *Y*‐axis the proportion of fragments with the corresponding fragment size. HCC samples are indicated in blue and HBV samples in red. (B) Box plots of the mean proportions of cfDNA fragments < 150 bp in all fragment sizes from 20 HCC samples with TF > 0.2, 43 HCC samples with 0 < TF ≤ 0.2, and 187 HBV samples. The mean proportion of cfDNA fragments < 150 bp among three groups was compared using one‐tail *t*‐tests. Orange line represents mean and whiskers represent range.

After CNV analysis, the size distribution of cfDNA of HBV‐HCC samples with TF > 0.2 and control samples with HBV infection was explored. The cfDNA of all samples showed a prominent peak at 167 bp in the size distribution plot for both HBV‐HCC and HBV samples (Fig. 2A). A distinct difference of fragment lengths was observed between cfDNA derived from HCC and HBV samples, in that cfDNA fragment lengths in HBV‐HCC samples were obviously enriched in shorter lengths (< 150 bp) than those in HBV samples (Fig. 2A). Box plot analysis showed that the proportions of cfDNA fragments < 150 bp in high‐TF (TF > 0.2; mean = 0.39, *P* = 7.37e‐07) and low‐TF (0 < TF ≤ 0.2) (mean = 0.27, *P* = 5.29e‐07) HCC samples were all significantly higher than in HBV samples (mean = 0.2; Fig. 2B). These results demonstrated that cfDNA fragments shorter than 150 bp could well detect the CNV markers, consistent with previous reports [20]. This feature enables us to explore further the utility of fragment size to enhance the detection of cancer using CNV aberrations as a biomarker.

### 3.3 ctDNA enrichment with cfDNA fragment selection increases TF and CNV signals

**Fig. 3:**
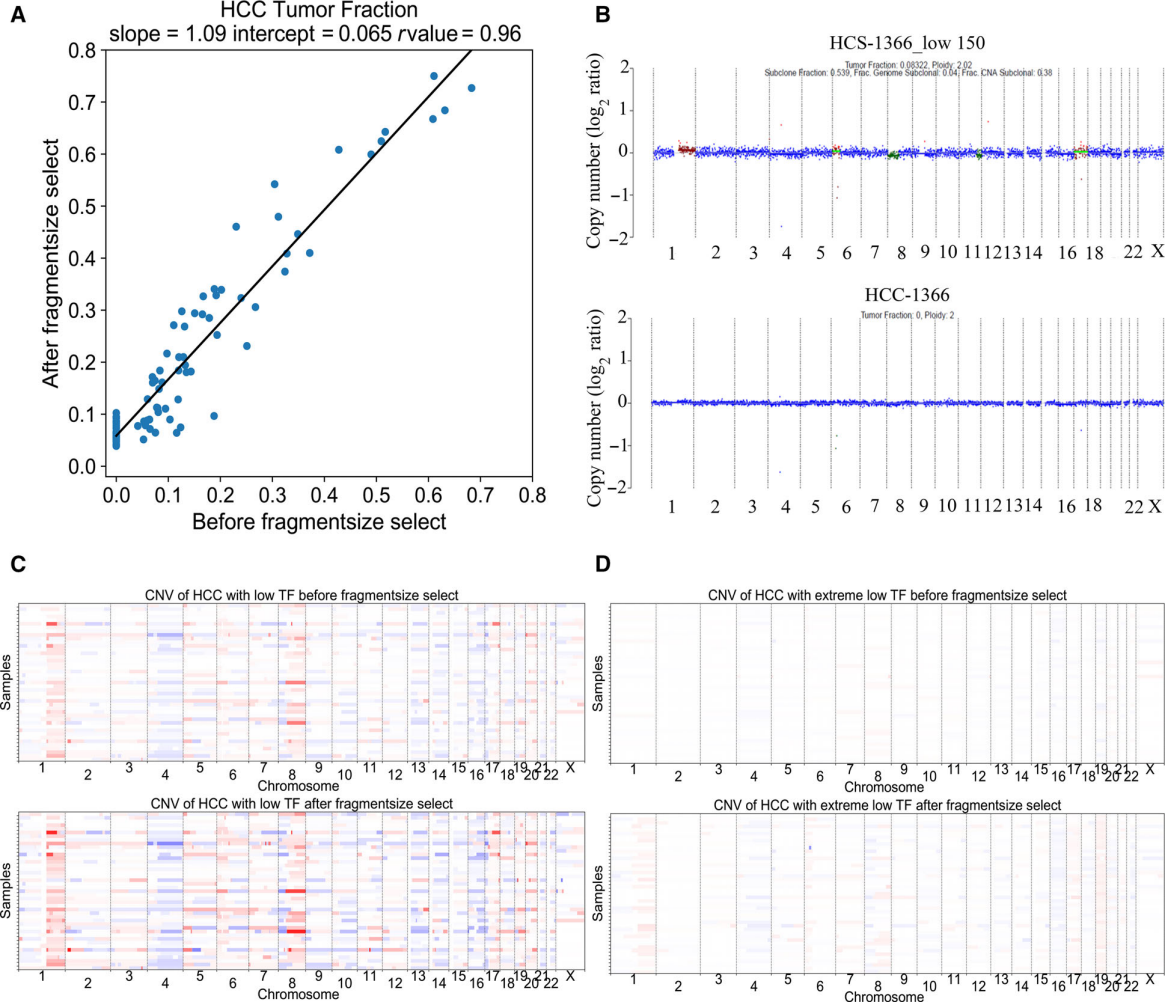
cfDNA fragment selection increases TF and CNV signals. (A) The effect of selecting fragments < 150 bp on the tumor purity of HCC samples using linear least‐squares regression. The tumor purity of each sample after size selection (*Y*‐axis) and before size selection (*X*‐axis) is shown. (B) CNV profiles of one HCC sample (HCC‐1366) before (bottom) and after fragment size enrichment (top). CNV amplifications are presented in red and CNV deletions in green. The darker the color is, the greater the amplitudes/deletions are. (C) CNV profiles of 43 HCC samples with ctDNA TF between 0 and 0.2 showing the CNV signal alterations before (top) and after selecting < 150 bp fragments (bottom). (D) CNV profiles of 44 HCC samples with low proportions of ctDNA in cfDNA (TF = 0) showing the CNV signal alterations before (top) and after < 150 bp fragment selection (bottom). CNV amplifications are presented in red and CNV deletions in blue. The darker the color is, the greater the amplitudes/deletions are.

In light of the above findings, we sought to evaluate whether the selection of cfDNA fragment size < 150 bp could enrich ctDNA fragments against the large background of cfDNA fragments. Before fragment selection, there were 63 HCC samples with TF > 0 and 134 HCC samples with TF = 0, and we observed a significantly increased number (*n* = 107) of HCC samples with estimated TF > 0 after selecting fragments of < 150 bp, corresponding to a 70% increase in detection rate. We further quantified the magnitude of ctDNA enrichment, and observed that the average tumor purity of cfDNA in HCC samples was enhanced by approximately 6.5% (Fig. 3A). For instance, prior to fragment size selection, the TF of one HCC sample (HCC‐1366) was estimated to be 0, and the CNV signal was undetectable. Of note, after the fragment size enrichment, the TF of this sample was enhanced to 0.08 and the CNV signal was readily detectable (Fig. 3B), demonstrating enriched ctDNA of HCC samples by utilizing the biomarker of fragment sizes.

For further analysis, we selected 43 HCC samples with ctDNA TF between 0 and 0.2 to investigate the strength of CNV signal alterations before and after < 150‐bp fragment selection. Although partial CNV signals could be observed prior to size selection, the CNV signals were markedly enhanced after fragment size enrichment (Fig. 3C). Interesting, we found that the CNV signals after size selection were highly consistent with the signal features in Fig. 1, representing the commonly occurring aberrant CNV we identified in this study, e.g. chr1, chr4 and chr8 (Fig. 3C). Based on the findings that the selection of < 150‐bp fragment size was able to improve TF of ctDNA, and the theoretical hypothesis that the strong CNV signal was associated with the high TF of ctDNA, we wondered whether the CNV signals in the samples with a lack of detectable CNV could be detected through increasing TF after fragment size selection. For this, the other 44 HCC samples with low ctDNA proportions in cfDNA (TF = 0) were chosen, and it was clear that CNV signals, including aberrant CNV in chr1, chr4 and chr8, emerged after fragment size selection (Fig. 3D). These findings demonstrated that by selecting > 150‐bp fragments of cfDNA, coupled with the CNV aberration biomarkers identified in this study, it was possible to enhance HCC‐specific CNV signals for cancer detection.

### 3.4 cfDNA end‐motif determination in HCC and HBV samples

**Fig. 4:**
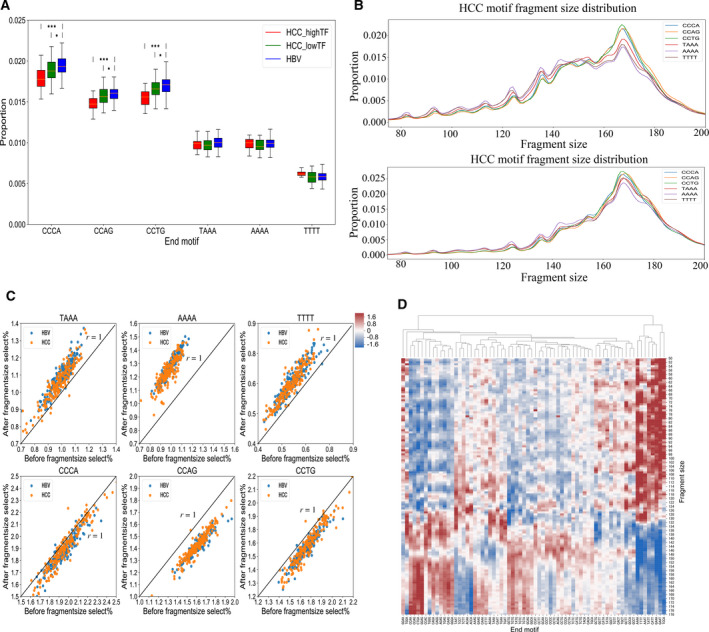
cfDNA end‐motif determination and the association of fragment sizes and end motifs in HCC (TF > 0, *n* = 63) and HBV samples (*n* = 187). (A) Box plot analysis of differential frequencies of six representative motifs (CCCA, CCTG, CCAG, TAAA, AAAA and TTTT) among high‐TF HCC (TF > 0.2; *n* = 20), low‐TF HCC (0 < TF ≤ 0.2; *n* = 43) and HBV groups (*n* = 187). The three groups were compared using one‐tail *t*‐tests. Orange line represents mean and whiskers represent range. *P < 0.05; ***P < 0.001. (B) Fragment size distributions of six motifs from HCC samples with TF > 0.2 (top) and HBV samples (bottom). (C) The effect of selection of fragments shorter than 150 bp on the proportion of motifs using linear least‐squares regression. The proportions of each motif in all cfDNA reads after size selection (*Y*‐axis) and before size selection (*X*‐axis) are shown. Dark blue dots represent HCC samples and orange dots HBV samples. (D) Heat map analysis of the association of fragment sizes and 70 motifs with a proportions > 0.005 from high‐TF HCC (TF > 0.2) samples. *X*‐axis represents the length of fragments and *Y*‐axis the proportion of the corresponding motif. Each matrix represents the mean proportion of reads of the corresponding end motif from all samples; the data were revised using the *z*‐score.

It has been reported that cfDNA fragmentation is a nonrandom course and there is a class of ctDNA signatures with preferred DNA ends (e.g. CCCA, CCTG, CCAG, TAAA, AAAA and TTTT) related to HCC [15, 21]. To investigate the end‐motif patterns in our data, we first counted the proportions of six representative end motifs (CCCA, CCTG, CCAG, TAAA, AAAA and TTTT), reported previously [15], in high‐TF (TF > 0.2) HCC, low‐TF (0 < TF ≤ 0.2) HCC, and HBV groups. As shown in Fig. 4A, the proportions of three motifs CCCA, CCTG and CCAG were higher in contrast to the other three motifs (TAAA, AAAA and TTTT) in all three groups. We observed significantly reduced proportions of the three motifs, i.e. CCCA (*P* = 5.42e−07), CCTG (*P* = 6.21e−08) and CCAG (*P* = 1.25e−07), in the high‐TF HCC group vs. the HBV group, which was consistent with a previous report [15], although the magnitudes of differences in our study were smaller than in the previous report. In particular, with the increase of cfDNA TF in HCC samples, the proportion of end motifs CCCA, CCTG and CCAG was significantly reduced (Fig. 4a). However, contrary to the previous report [15], we did not observe significant differences in the proportions of three motifs (TAAA, AAAA and TTTT) in HCC compared with HBV samples, regardless of increasing the TF of the HCC samples. These data demonstrated that the end motifs CCCA, CCTG and CCAG were HCC‐associated cfDNA preferred ends, whereas it was inconclusive whether TAAA, AAAA or TTTT was associated with ctDNA fragment ends; clarification of this requires future investigation.

**Fig. 5:**
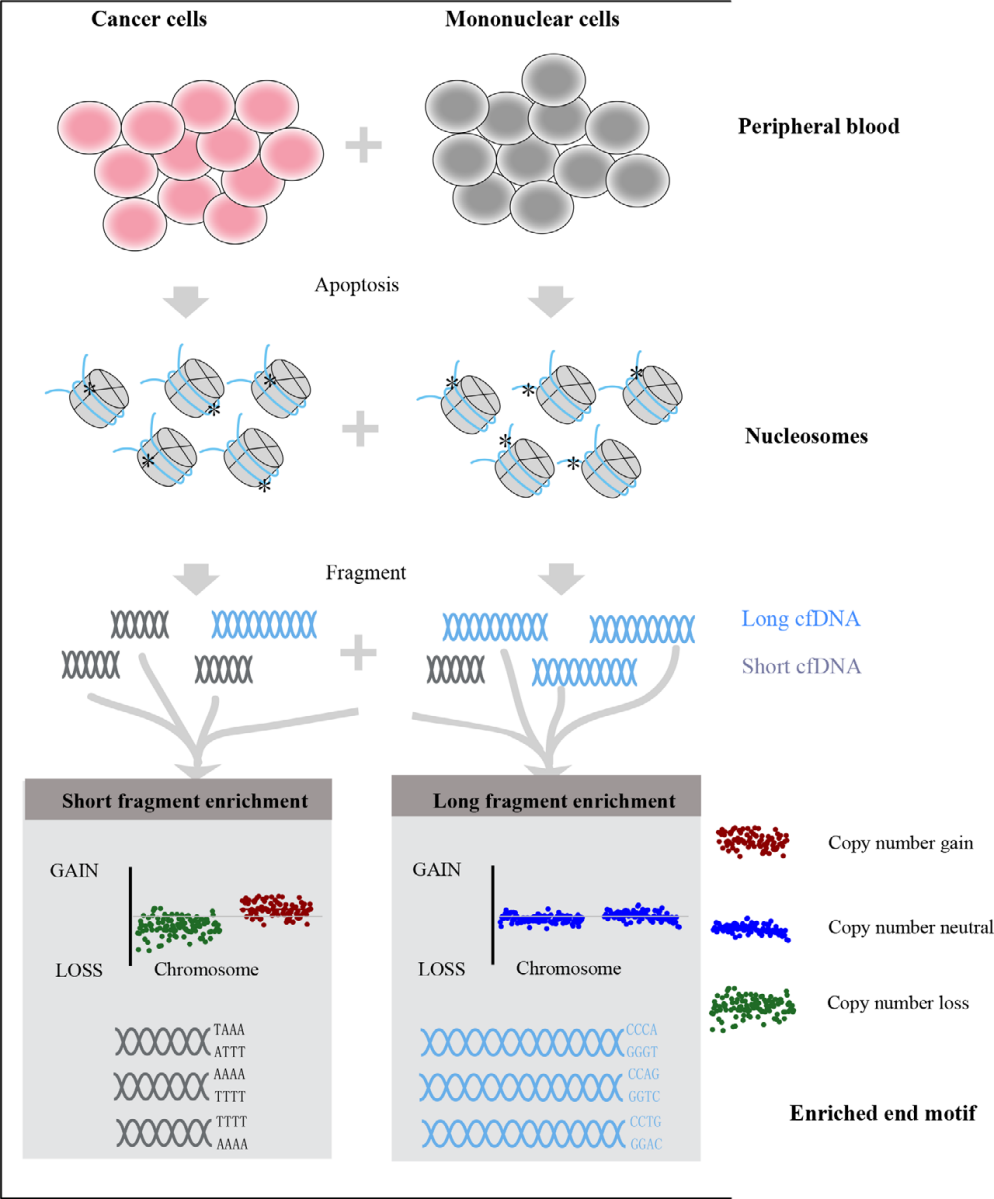
The inherent coupling of cfDNA fragment size and end motifs which might be associated with cfDNA cleavage process, in both HCC and HBV samples, as a ubiquitous mechanism.

Having established that both fragment sizes and end motifs are characteristics of ctDNA, and realizing that both may be involved in the ctDNA cleavage process, we wondered whether the two characteristics are independent events. To this end, we investigated the fragment size distribution of cfDNA sequencing reads separately for reads containing each of these six end motifs. We found that the fragment size distributions were different for different end motifs for both HCC and HBV samples (Fig. 4B). In particular, the difference was more profound in fragment sizes < 150 bp, corresponding to the fragment sizes that were strongly associated with ctDNA (Fig. 4B). Among these six motifs, reads with the end motif of AAAA were enriched in shorter reads of < 150 bp, whereas reads with the CCAG motif were longer (Fig. 4B). We found that the fragment size distribution of reads with these end motifs in HBV samples was similar to that in HCC samples (Fig. 4B), indicating that the association of fragment size with end motifs was not unique to HCC. To quantify further the magnitude of the association for different motifs, we investigated the effects of fragment size selection on the changes in the proportion of these six end motifs for both HCC and HBV samples, respectively. The results demonstrated that the proportions of end motifs CCCA, CCAG and CCTG were consistently decreased in HCC and HBV samples after fragment size selection of < 150 bp, compared with that before fragment size selection (Fig. 4C). Conversely, the proportions of end motifs TAAA, AAAA and TTTT were consistently increased in HCC and HBV after fragment size selection (Fig. 4C). These findings are consistent with results in Fig. 4(A,B) and could be explained by a larger fragment size of the end motifs CCCA, CCAG and CCTG than of the end motifs TAAA, AAAA and TTTT. Of particular importance, we observed that the change in the pattern of the proportions of these six end motifs in HCC and HBV samples after size selection, were largely indistinguishable, suggesting that the coupling of fragment size and end motifs is likely to be a general mechanism involved in the cfDNA cleavage process, rather than a process specific to HCC (Fig. 5).

In addition to the above six representative 4‐mer end motifs, we were interested in comprehensively investigating the proportions of all 256 4‐mer cfDNA end motifs in reads of HCC and HBV samples before and after fragment size selection. We found that the proportion of 139 4‐mer cfDNA end motifs was significantly changed after fragment size selection, and the changes were consistent in both HCC and HBV samples, compared with those prior to fragment size selection (Fig. S1), indicating the complex coupling of fragment sizes and end motifs. Among the significantly associated motifs, the proportions of 69 end motifs were significantly increased and the proportions of 70 end motifs significantly decreased after fragment size selection in both HCC and HBV samples (Table S1). The top 10 most significant (smallest *P*‐values) end motifs are listed in Table 2. Interesting, 80% (8/10) of the top 10 end motifs (e.g. AAAA, GAAT, GAAA and TCCA) were shared in both HCC and HBV samples. We did not, however, find changes in the proportion of any end motif in HCC samples after fragment size selection that differed from that in HBV samples.

**Table 2:** Top 10 most significant (smallest *P*‐values) end motifs showing proportion changes in HCC and HBV samples after fragment size selection.

Motif	Mean	Difference	*P*‐value
HCC			
TTCC	0.001734	0.759349	5.50E−136
GAAT	0.005042	0.299973	3.59E−110
ATGG	0.001753	0.496929	1.24E−104
GAAA	0.007313	0.203002	1.87E−98
AAAA	0.009739	0.273082	4.14E−92
TCCA	0.005373	0.181188	1.57E−80
AATG	0.003325	0.272515	1.90E−80
ATTC	0.001841	0.375516	5.94E−77
AATT	0.002967	0.280476	8.92E−71
GGAC	0.003352	−0.10491	4.27E−65
HBV			
GAAA	0.00734	0.216998	8.98E−153
GAAT	0.005054	0.314193	5.12E−152
TTCC	0.001652	0.82927	7.68E−141
ATGG	0.001693	0.531796	6.93E−140
AAAA	0.009874	0.289954	7.95E−117
CGTA	0.000953	−0.13613	3.05E−116
TCCA	0.005338	0.187821	3.17E−113
GATT	0.003373	0.228652	7.96E−110
ATTC	0.001849	0.380833	3.90E−107
AATG	0.003363	0.27518	5.70E−107

Taking into consideration the nonrandomness of cfDNA fragmentation, we wondered whether there were some finer‐scale associations between fragment size of cfDNA and end motifs. We therefore explored the clustering of end motifs and fragment sizes. A total of 70 motifs with proportions > 0.005 in both HCC and HBV samples were selected for this analysis. The heat map demonstrated that there were groups of motifs that were associated with shorter fragment sizes, whereas some motifs were clustered with longer fragments. For example, AAAA, TTTT, AAAT, TATT, CATT, GAAA, GAAT and TCCA were consistently coupled with fragments shorter than 150 bp in both HCC and HBV samples, whereas end motifs (e.g. CCAA, CCAG, CTGA, GGAG, CCAC, TGGG, CAGA, CAGG, AGAG, TGAG, CAAG and GGGA) were consistently coupled with fragments larger than 150 bp in both HCC and HBV samples (Figs 4D, 5, S2 and S3). The strong coupling of groups of motifs with either short or long fragments suggests a mechanism by which these motifs participate in the fragmentation of cfDNA.

## 4 Discussion

In this study, we comprehensively investigated the fragmentomic features of cfDNA derived from WGS data of HCC and HBV, as well as the CNV aberrations that could be used for cancer detection. We identified commonly occurring CNV alterations in HCC and, in particular, we identified five typical CNV as biomarkers for HCC detection. We found the positive rate of the five CNV was higher in HCC samples with high TF than in those with low TF, and abnormal CNV might be considered a preclinical signal to assist in early detection of HCC. In addition, our result showed that the proportion of fragments with a length < 150 bp was obviously higher in HCC samples with high‐TF than in low‐TF and HBV samples, and selection of < 150‐bp length not only enhanced the TF to improve the clinical utility of ctDNA detection but also provided a new strategy for detecting CNV more accurately for HCC samples with lower CNV signals. For motif analyses, three 4‐mer end motifs (CCCA, CCTG and CCAG) were identified as preferred end motifs of HCC, but we were not able to replicate the HCC‐associated motifs (TAAA, AAAA and TTTT) reported previously [15]. Our study is the first, to our knowledge, to investigate the association of fragment sizes and 4‐mer end motifs in HCC and HBV, and to identify a group of 139 end motifs that are significantly associated with fragment size. In addition, we found that the size characteristics of these end motifs in HCC samples were similar to those in HBV samples.

Copy number variations detected by next‐generation sequencing, account for important types of genomic abnormalities in cancers, from sub‐microscopic events to complete chromosomal aneuploidies [22, 23]. Several very common chromosomal arm alterations in both HCC and HBV‐infected HCC tissue specimens have been revealed, including loss of chr1p, chr8p and chr17p, and gain in chr1q and chr8q [24, 25]. Interestingly, chromothripsis, a phenomenon with a large number of rearrangements clustered in a chromosomal region, may drive chr 1q and 8q amplifications to contribute to hepatocarcinogenesis [26]. In addition, significant changes in CNV (gain in chr1q, chr7q, and chr19q, and loss of chr1p, chr9q and chr14q) have been detected in blood samples of HCC with chronic liver diseases [27]. Consistently, in this work, some typical CNV changes comprised of loss of chr1p, chr4q and chr8p, and gain in chr1q and chr8q were frequently detected in HBV‐HCC blood samples. Of note, two of 187 HBV subjects exhibited abnormal CNV and were diagnosed with HCC several months later. It is possible that abnormal CNV may be considered a preclinical signal for early detection of HCC. Notably, our results regarding the landscape of genomic CNV of HCC based on liquid biopsy were very consistent with the corresponding results derived from tissue‐based analyses by Zhang *et al*. [25], suggesting that our data are reliable and WGS sequencing of cfDNA is able to detect CNV harbored in ctDNA. As the five CNV aberrations were derived from cfDNA in HCC samples, the high coverage (˜ 95% of cfDNA samples from HCC patients) of these CNV biomarkers indicates promising targets for early detection of HCC.

In our work, the cfDNA size distribution plot of HCC and HBV samples showed a prominent peak at 167 bp. This length is in close proximity to the length of DNA wrapped around a nucleosome and its linker [28]. This fragmentation pattern may have resulted from liberation of cfDNA via cell apoptosis and necrosis, during which histone complex binding to nuclear DNA acts as a main degradation type to protect DNA from cleavage [29, 30]. In addition, we found that cfDNA fragments < 150 bp were more likely to be enriched in HCC samples than in HBV samples, which was similar to previous findings in animal and human cancers [14, 31]. Of note, we found the proportion of lengths < 150 bp was obviously higher in HCC samples with high‐TF than in HCC samples with low‐TF and HBV samples, supported by findings that shorter fragments were enriched in HCC patients with higher levels of ctDNA [32]. These findings motivated us to select cfDNA fragment size < 150 bp to enrich ctDNA of HCC samples and to observe the CNV signals. As a result, tumor purity of cfDNA of HCC samples was enhanced ˜ 6.5% by selecting the fragment size of < 150 bp. Similarly, it has been reported that the selection of 90‐ to 150‐bp fragment size may more than double the enrichment of tumor DNA in > 95% of cases [20], and on average 44% more CNA was detectable in cancer patients after selecting fragments with sizes up to 142 bp (± 15 bp) [33].

There is evidence that cfDNA has a higher predilection for specific end‐motif sequence as a result of being cut at specific genomic regions or elements [34]. A class of cfDNA with preferred ends was found to be selectively associated with fetal‐ or maternal‐derived DNA, and the ratio of fetal preferred ends to those with maternal preferred ends in maternal plasma presents a correlation with the fetal DNA fraction [35]. Similarly, another key observation by Jiang *et al*. [15]of DNA end characteristics in HCC was that there are 5.4 million HCC‐associated cfDNA preferred ends and 4.4 million preferred ends shared in HCC and chronic hepatitis B patients. In addition, those authors suggested that the abundance of HCC‐related DNA is associated with DNA TF. Recently, Jiang *et al*. discovered a group of 4‐mer end motifs showing significant differences between HCC and non‐HCC subjects, in which the frequencies of the motifs CCCA, CCAG and CCTG were significantly decreased in HCC subjects, and the frequencies of TAAA, AAAA and TTTT were significantly increased in HCC subjects, as compared with non‐HCC subjects [21]. However, in our work, no significant difference of frequencies of motifs (TAAA, AAAA and TTTT) among high‐TF HCC, low‐TF HCC and HBV groups was detected. Although we found that the lower proportion of three end motifs (CCCA, CCTG and CCAG) was significantly related to HBV‐HCC and higher cfDNA TF, the magnitude of the differences among groups was not as dramatic as the results of Jiang *et al*. [21]. These differences might be caused by differences in the subjects studied. We only collected HCC with HBV infection samples, whereas Jiang *et al*. focused on HCC subjects without HBV infection. HBV‐infected individuals are at high risk of developing HCC and may also harbor driver mutations prevalent in HCC. Collectively, our results showed that the motifs CCCA, CCTG and CCAG may be HCC‐related preferred end motifs. Future research is still needed to investigate the associations of motifs TAAA, AAAA and TTTT with HCC.

There is evidence to show short and long plasma DNA molecules are associated with different preferred DNA end sites [36]. In our work, we investigated whether there were some motifs among the 256 4‐mer end motifs related to fragment size specific to HCC. We found that the proportion of 69 4‐mer end motifs (e.g. TAAA, AAAA and TTTT) were significantly increased and 70 4‐mer end motifs (e.g. CCCA, CCTG and CCAG) were significantly decreased in both HCC and HBV samples after the selection of a fragment size of < 150 bp. Notably, the proportion changes of any end motif after fragment size selection in HCC samples were not found to be different from those in HBV samples. Clustering analysis revealed a more pervasive coupling of end motifs with fragment sizes (Figs 4D and 5). These findings suggest that end motifs may be inherently coupled with fragment sizes in both HCC and HBV, as a ubiquitous mechanism (Fig. 5). Further investigations are needed to dissect the mechanisms behind this coupling to reveal fine‐scale characteristics of fragmentomic features in order to facilitate more powerful detection of HCC, as well as other cancer types.

## 5 Conclusions

We identified representative abnormal CNV alterations related to HCC and found that selecting fragment sizes shorter than 150 bp was an effective strategy for detecting CNV more accurately and for improving the clinical utility of ctDNA detection of HCC samples. The study discovered strong coupling of end motifs with fragment sizes, and revealed similar fragment size characteristics of 4‐mer end motifs between HCC and HBV subjects.

## Conflict of interest

WYZ, HPL, BX, JYY and QZ are employees of Oriomics Biotech Inc. No other disclosures of interest were reported.

## Author contributions

CJ, XLi, WYZ, JL and JX conceived and supervised the study. LS, JY and QZ conducted data collection. WZ, YL and BX participated in the data analysis. CJ and XLi wrote the first draft of the manuscript. JL, JX, XuG, XiG, HL, GW, DB and SW provided the main edits of the manuscript, and JL and JX had responsibility for final content. All authors read and approved the final manuscript.

## Supporting information

**Fig. S1**. The effect of selection of fragments < 150 bp on the changes in proportion of 256 4‐mer end motifs using linear least‐squares regression. The proportions of each motif in all cfDNA reads after size selection (*Y*‐axis) and before size selection (*X*‐axis) are shown. Dark blue dots represent HCC samples and orange dots HBV samples.

**Fig. S2**. Heat map analyses of the associations of fragment size and 70 motifs with proportions > 0.005 from low‐TF HCC samples (0 < TF ≤ 0.2). *X*‐axis represents the length of fragment and *Y‐*axis the proportion of corresponding motifs. Each matrix represents the mean proportion of reads of the corresponding end motif from all samples; the data were revised using the *z‐*score.

**Fig. S3**. Heat map analyses of the association of fragment size and 70 motifs with proportions > 0.005 from HBV samples. *X*‐axis represents the length of fragment and *Y‐*axis represents the proportion of corresponding motifs. Each matrix represents the mean proportion of reads of the corresponding end motif from all samples; and the data were revised using the *z‐*score.

**Table S1**. The significance of changes in proportion of 139 4‐mer cfDNA end motifs in HCC and HBV samples, respectively, after fragment size selection.

## Data Availability

The data that support the findings of this study are openly available in BIG Sub at https://bigd.big.ac.cn/gsub/, accession number subCRA00289, and other data generated during this study are included in Supporting Information files.
