# Supplementary figures and images for: Characterization of fragment sizes, copy number aberrations and 4‐mer end motifs in cell‐free DNA of hepatocellular carcinoma for enhanced liquid biopsy‐based cancer detection
Source: Mol Oncol. 2021 Jul 16;15(9):2377–89. doi: 10.1002/1878-0261.13041 (PMC8410516; doi:10.1002/1878-0261.13041)

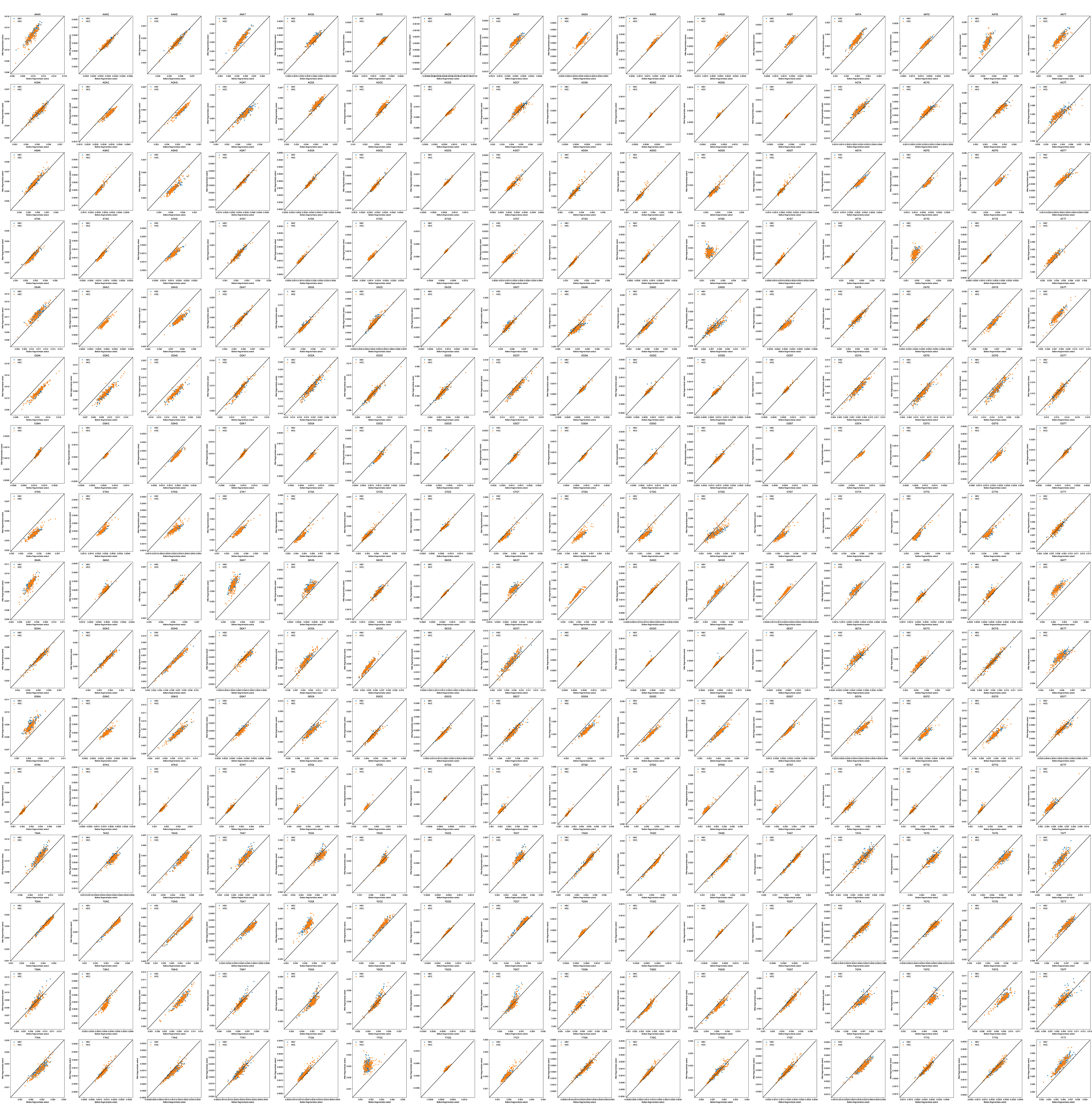

Supplement: Supplementary file 1 — Fig. S1. The effect of selection of fragments < 150 bp on the changes in proportion of 256 4‐mer end motifs using linear least‐squares regression. The proportions of each motif in all cfDNA reads after size selection (Y‐axis) and before size selection (X‐axis) are shown. Dark blue dots represent HCC samples and orange dots HBV samples. [file MOL2-15-2377-s003.tif]

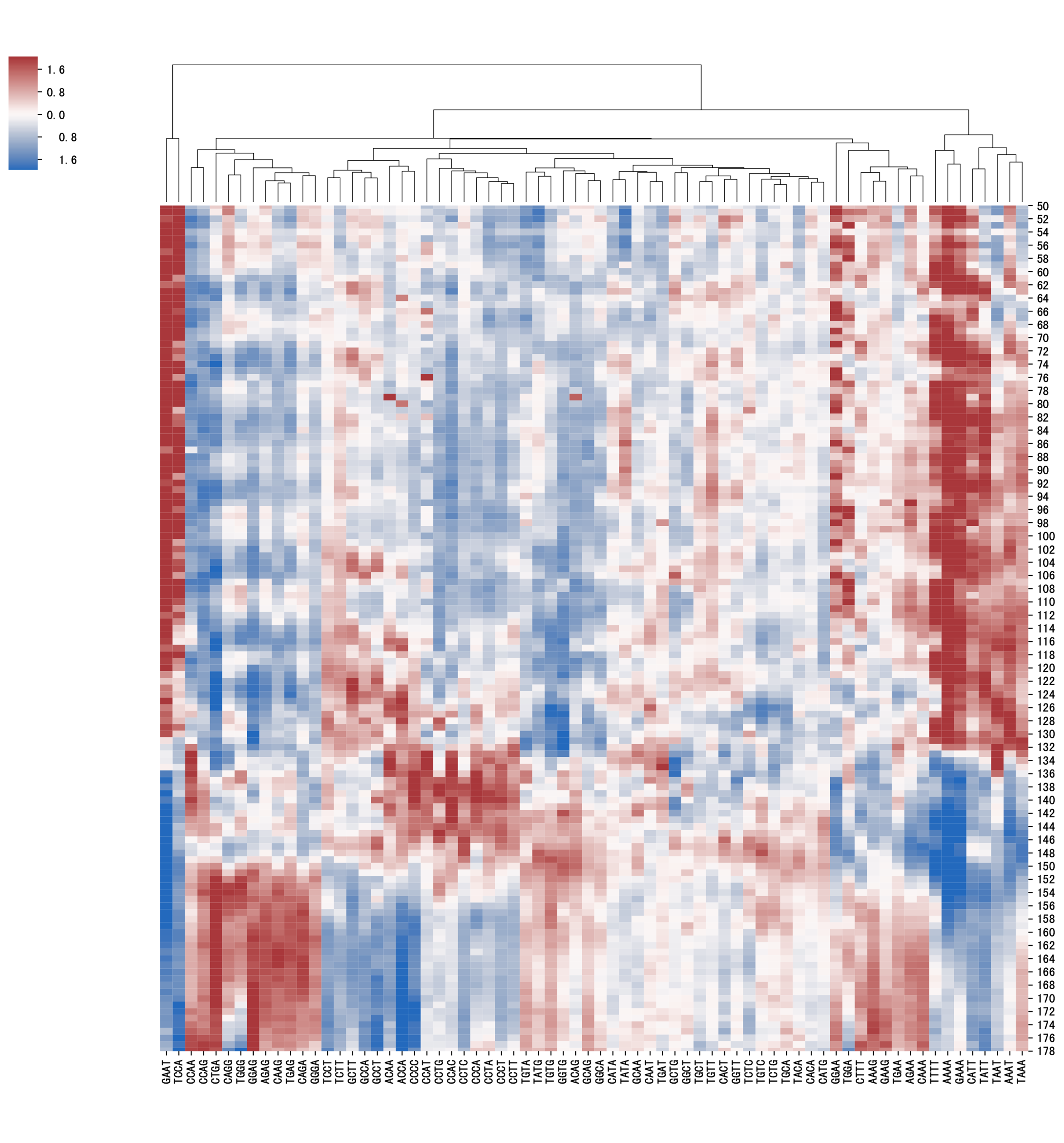

Supplement: Supplementary file 2 — Fig. S2. Heat map analyses of the associations of fragment size and 70 motifs with proportions > 0.005 from low‐TF HCC samples (0 < TF ≤ 0.2). X‐axis represents the length of fragment and Y‐axis the proportion of corresponding motifs. Each matrix represents the mean proportion of reads of the corresponding end motif from all samples; the data were revised using the z‐score. [file MOL2-15-2377-s001.tif]

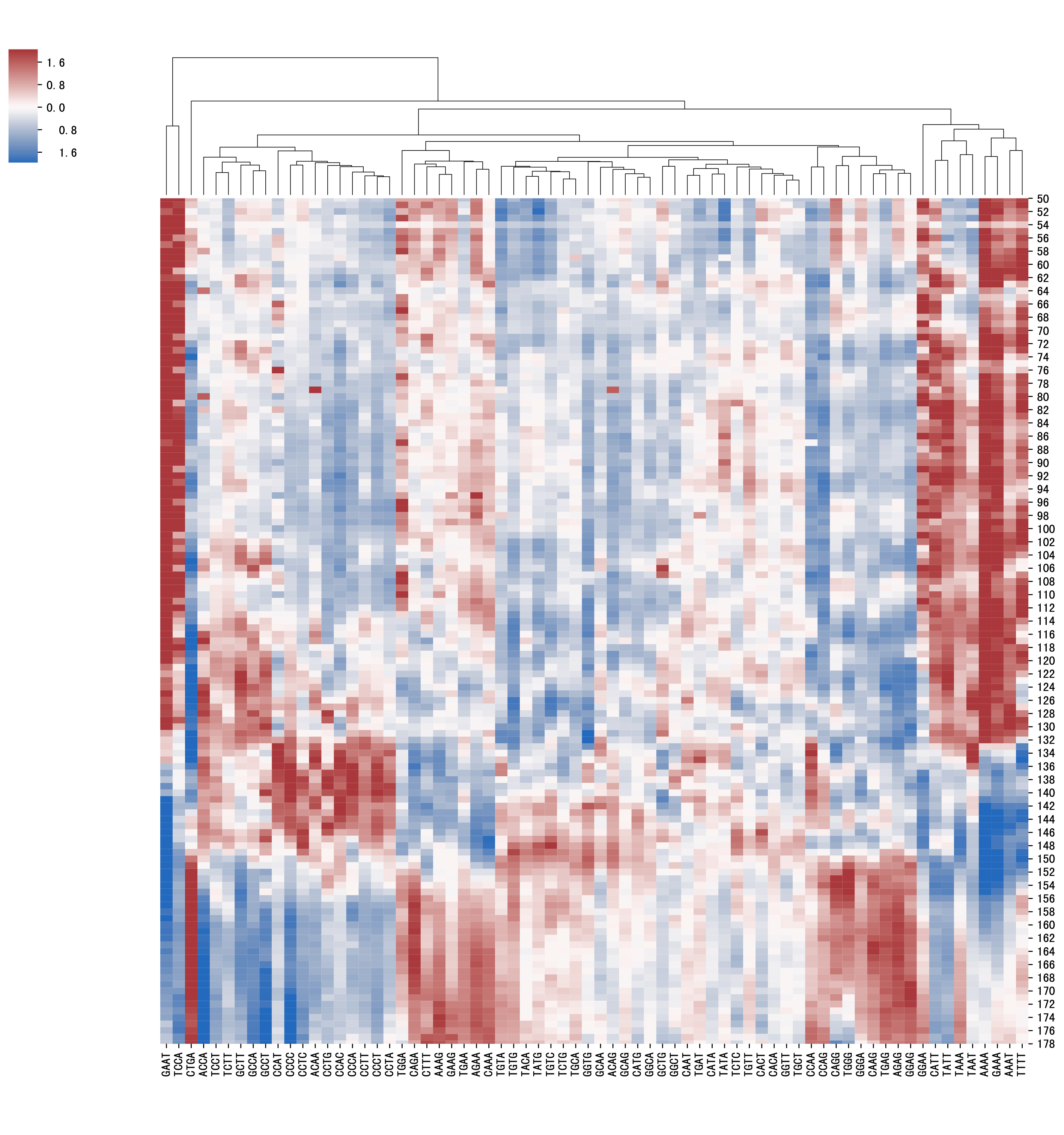

Supplement: Supplementary file 3 — Fig. S3. Heat map analyses of the association of fragment size and 70 motifs with proportions > 0.005 from HBV samples. X‐axis represents the length of fragment and Y‐axis represents the proportion of corresponding motifs. Each matrix represents the mean proportion of reads of the corresponding end motif from all samples; and the data were revised using the z‐score. [file MOL2-15-2377-s004.tif]
