# Supplementary material for: Characterization of fragment sizes, copy number aberrations and 4‐mer end motifs in cell‐free DNA of hepatocellular carcinoma for enhanced liquid biopsy‐based cancer detection
Source: Mol Oncol. 2021 Jul 16;15(9):2377–89. doi: 10.1002/1878-0261.13041 (PMC8410516; doi:10.1002/1878-0261.13041)
Supplement: Supplementary file 4 — Table S1. The significance of changes in proportion of 139 4‐mer cfDNA end motifs in HCC and HBV samples, respectively, after fragment size selection. [file MOL2-15-2377-s002.docx]

**Table S1** The significance of proportions changes of 139 4-mer cfDNA end motifs in HCC and HBV samples after fragment size selection, respectively.

| **Motif** | **hcc_mean** | **hcc_diff** | **hcc_pvalue** | **Motif** | **hbv_mean** | **hbv_diff** | **hbv_pvalue** |
| --- | --- | --- | --- | --- | --- | --- | --- |
| TTCC | 0.001734 | 0.759349 | 5.50E-136 | GAAA | 0.00734 | 0.216998 | 8.98E-153 |
| GAAT | 0.005042 | 0.299973 | 3.59E-110 | GAAT | 0.005054 | 0.314193 | 5.12E-152 |
| ATGG | 0.001753 | 0.496929 | 1.24E-104 | TTCC | 0.001652 | 0.82927 | 7.68E-141 |
| GAAA | 0.007313 | 0.203002 | 1.87E-98 | ATGG | 0.001693 | 0.531796 | 6.93E-140 |
| AAAA | 0.009739 | 0.273082 | 4.14E-92 | AAAA | 0.009874 | 0.289954 | 7.95E-117 |
| TCCA | 0.005373 | 0.181188 | 1.57E-80 | CGTA | 0.000953 | -0.13613 | 3.05E-116 |
| AATG | 0.003325 | 0.272515 | 1.90E-80 | TCCA | 0.005338 | 0.187821 | 3.17E-113 |
| ATTC | 0.001841 | 0.375516 | 5.94E-77 | GATT | 0.003373 | 0.228652 | 7.96E-110 |
| AATT | 0.002967 | 0.280476 | 8.92E-71 | ATTC | 0.001849 | 0.380833 | 3.90E-107 |
| GGAC | 0.003352 | -0.10491 | 4.27E-65 | AATG | 0.003363 | 0.27518 | 5.70E-107 |
| GATT | 0.003339 | 0.217683 | 8.04E-65 | CTGA | 0.005468 | -0.16922 | 1.56E-103 |
| GGAA | 0.008186 | 0.079659 | 5.69E-60 | AAGA | 0.003603 | 0.156384 | 2.13E-93 |
| AAGA | 0.003602 | 0.144238 | 7.98E-58 | AATT | 0.003036 | 0.292651 | 2.85E-93 |
| CAAG | 0.007706 | -0.06951 | 2.12E-52 | CTAA | 0.004456 | -0.17593 | 7.46E-92 |
| CTGA | 0.005535 | -0.15929 | 2.15E-52 | GTCC | 0.001055 | 0.19012 | 1.75E-91 |
| CTAG | 0.003091 | -0.16596 | 1.10E-48 | GGAC | 0.003332 | -0.10471 | 1.54E-85 |
| CATT | 0.007431 | 0.150218 | 4.31E-48 | CATT | 0.007492 | 0.15649 | 6.86E-85 |
| CTAA | 0.004444 | -0.16492 | 5.47E-44 | TGTG | 0.009056 | -0.10622 | 5.46E-80 |
| GGAG | 0.009587 | -0.11122 | 1.04E-42 | GTGT | 0.001689 | 0.136598 | 5.58E-78 |
| TGTC | 0.005734 | -0.07706 | 9.08E-42 | GGAA | 0.00816 | 0.088274 | 2.04E-77 |
| AGTG | 0.004056 | -0.07896 | 4.59E-41 | CTAC | 0.002311 | -0.11836 | 2.21E-75 |
| CGTA | 0.000939 | -0.13059 | 1.68E-40 | CCAA | 0.012211 | -0.09992 | 2.21E-72 |
| CCAG | 0.016097 | -0.11215 | 1.23E-38 | CTAG | 0.003073 | -0.17376 | 4.64E-71 |
| AATA | 0.002906 | 0.199023 | 3.15E-38 | GGTG | 0.007779 | -0.11891 | 3.25E-70 |
| CGTG | 0.002286 | -0.1124 | 1.40E-33 | GGAG | 0.009607 | -0.11492 | 1.21E-68 |
| GGTG | 0.007789 | -0.10946 | 1.84E-33 | AGTG | 0.00405 | -0.09097 | 2.61E-68 |
| TGTG | 0.008907 | -0.10092 | 2.56E-33 | GACT | 0.002997 | 0.090268 | 1.09E-67 |
| CGTC | 0.001096 | -0.10629 | 1.83E-32 | CTTA | 0.004209 | -0.13381 | 3.02E-66 |
| GACT | 0.00297 | 0.080191 | 3.87E-32 | CCAG | 0.01604 | -0.11981 | 1.09E-64 |
| CGAC | 0.000595 | -0.09174 | 7.86E-31 | CAAC | 0.003822 | -0.05889 | 1.36E-62 |
| GGTC | 0.003827 | -0.07103 | 2.33E-30 | GAGA | 0.003788 | 0.146161 | 2.71E-60 |
| AAAT | 0.005554 | 0.127441 | 2.72E-29 | AAGT | 0.001906 | 0.103181 | 4.94E-60 |
| AACT | 0.002512 | 0.093809 | 3.07E-29 | CTAT | 0.003341 | -0.14126 | 5.71E-60 |
| AAGT | 0.001896 | 0.094118 | 3.29E-29 | CAAG | 0.007707 | -0.07065 | 4.28E-59 |
| CAAC | 0.003845 | -0.05629 | 2.30E-28 | ACAG | 0.005419 | -0.0816 | 4.40E-58 |
| TGAG | 0.009255 | -0.09981 | 5.07E-28 | TGTC | 0.005752 | -0.07917 | 6.18E-57 |
| GACA | 0.004195 | 0.058733 | 8.47E-28 | CTTG | 0.004344 | -0.07951 | 1.19E-56 |
| AGAG | 0.005246 | -0.08919 | 1.79E-27 | TGAG | 0.009299 | -0.09843 | 6.83E-56 |
| CCAC | 0.009602 | -0.07929 | 1.30E-26 | CGTG | 0.002293 | -0.11668 | 1.40E-55 |
| AAGG | 0.002534 | 0.085277 | 1.56E-26 | AATA | 0.002984 | 0.20587 | 3.18E-53 |
| CCAA | 0.012159 | -0.09296 | 1.99E-26 | AAGG | 0.002505 | 0.090922 | 4.04E-52 |
| CGAG | 0.00191 | -0.10324 | 3.89E-26 | AGAG | 0.005206 | -0.09451 | 6.83E-52 |
| CTAT | 0.003314 | -0.13653 | 4.78E-26 | TGAC | 0.004067 | -0.07073 | 1.14E-50 |
| GGGA | 0.00567 | -0.06577 | 6.26E-26 | CCAC | 0.009527 | -0.08613 | 3.79E-49 |
| CGCA | 0.00099 | -0.10122 | 6.27E-26 | GGTA | 0.004198 | -0.09272 | 1.57E-46 |
| TATT | 0.008209 | 0.137586 | 7.31E-26 | GACA | 0.004211 | 0.064028 | 2.26E-46 |
| CTTA | 0.00417 | -0.12176 | 5.15E-25 | GTGG | 0.001666 | 0.141874 | 2.49E-46 |
| GGTA | 0.004145 | -0.08815 | 6.44E-25 | GGTC | 0.003791 | -0.07747 | 4.35E-45 |
| AATC | 0.001763 | 0.081599 | 1.21E-23 | ACAC | 0.003377 | -0.06933 | 8.14E-45 |
| TTTT | 0.005724 | 0.13108 | 3.44E-23 | CGTC | 0.001082 | -0.1114 | 1.80E-44 |
| GAGA | 0.00383 | 0.132914 | 6.04E-23 | AGTC | 0.002239 | -0.07535 | 1.13E-43 |
| GATA | 0.002462 | 0.10642 | 9.36E-23 | CTTC | 0.004022 | -0.06718 | 1.46E-43 |
| TGAC | 0.004097 | -0.0712 | 2.83E-22 | AACT | 0.002562 | 0.09959 | 1.97E-43 |
| CTAC | 0.002352 | -0.10573 | 6.87E-22 | TATT | 0.008397 | 0.15195 | 3.91E-43 |
| GCTT | 0.005799 | 0.08034 | 6.96E-22 | GTCA | 0.001678 | 0.097413 | 5.12E-43 |
| ATAG | 0.001677 | -0.11789 | 2.21E-19 | GTCT | 0.002078 | 0.102243 | 6.87E-42 |
| ACAG | 0.005316 | -0.07594 | 4.42E-19 | TTCT | 0.001802 | 0.147685 | 2.03E-41 |
| GTCC | 0.0011 | 0.180721 | 7.61E-19 | TCGA | 0.000274 | 0.296793 | 1.22E-40 |
| ACAC | 0.003324 | -0.06272 | 8.54E-19 | AAAT | 0.005643 | 0.134406 | 3.73E-40 |
| AGTC | 0.00226 | -0.06508 | 4.18E-18 | CTGT | 0.004278 | -0.08996 | 7.10E-40 |
| CGGT | 0.000734 | -0.09813 | 9.83E-18 | GCTT | 0.005915 | 0.082793 | 1.45E-39 |
| TCGA | 0.000298 | 0.254291 | 5.17E-17 | CGAC | 0.000592 | -0.09161 | 7.40E-38 |
| CTTG | 0.004399 | -0.06732 | 1.54E-16 | TTCG | 0.000211 | 0.228882 | 9.10E-38 |
| GGGC | 0.003397 | -0.10402 | 1.39E-15 | GATA | 0.002507 | 0.110819 | 1.83E-37 |
| GAGT | 0.002108 | 0.114658 | 1.41E-15 | GGGA | 0.005634 | -0.06458 | 4.83E-36 |
| GGCA | 0.007445 | -0.05532 | 1.81E-15 | GAGT | 0.002094 | 0.126055 | 1.78E-35 |
| GCCC | 0.004905 | 0.118187 | 4.33E-15 | CTCA | 0.004553 | -0.07825 | 2.73E-35 |
| GTTT | 0.004503 | 0.099825 | 2.05E-14 | ATCC | 0.001049 | 0.092031 | 9.69E-35 |
| GTGT | 0.001734 | 0.139263 | 1.55E-13 | ACGG | 0.000219 | 0.168695 | 1.31E-34 |
| CTGT | 0.004364 | -0.08285 | 2.88E-13 | GGGC | 0.003345 | -0.1095 | 2.70E-34 |
| TCAT | 0.004656 | -0.05561 | 4.72E-13 | CTGC | 0.003513 | -0.09778 | 3.16E-32 |
| TTCG | 0.000223 | 0.193791 | 6.82E-13 | CATG | 0.006668 | -0.04129 | 1.28E-30 |
| CGCC | 0.001484 | -0.08652 | 7.83E-13 | ATAG | 0.001691 | -0.12241 | 3.85E-30 |
| GGAT | 0.004618 | -0.03374 | 3.26E-12 | CGCA | 0.000974 | -0.09997 | 6.26E-30 |
| TGTA | 0.006779 | -0.06185 | 4.23E-12 | TATG | 0.005431 | -0.07232 | 1.87E-29 |
| CAGT | 0.004567 | -0.03586 | 5.67E-12 | GCCC | 0.004862 | 0.124197 | 1.49E-28 |
| TAAT | 0.006597 | 0.070748 | 8.71E-12 | AATC | 0.00179 | 0.080278 | 3.63E-28 |
| TAAA | 0.009746 | 0.07754 | 8.77E-12 | CGAG | 0.001918 | -0.09676 | 1.05E-27 |
| TATG | 0.005246 | -0.07336 | 8.84E-12 | CGGT | 0.000719 | -0.1029 | 2.84E-26 |
| AGTA | 0.002645 | -0.05817 | 1.13E-11 | TTTT | 0.005842 | 0.128862 | 2.55E-25 |
| TAAG | 0.004979 | -0.07333 | 1.18E-11 | GCGT | 0.000257 | 0.159808 | 2.69E-24 |
| GAAC | 0.002895 | 0.035543 | 1.39E-11 | ACGA | 0.000232 | 0.070068 | 3.85E-24 |
| CATG | 0.006676 | -0.03701 | 1.69E-11 | TGTA | 0.006916 | -0.06172 | 4.89E-24 |
| TAAC | 0.003447 | -0.05712 | 1.88E-11 | TACT | 0.004613 | 0.067129 | 5.64E-24 |
| TACT | 0.004527 | 0.059552 | 3.66E-11 | GGCA | 0.007454 | -0.05557 | 1.62E-23 |
| TTCT | 0.001871 | 0.138976 | 4.87E-11 | TAAA | 0.009972 | 0.093988 | 2.49E-23 |
| ATCC | 0.001066 | 0.092341 | 6.06E-11 | CAAA | 0.010821 | 0.04591 | 6.35E-23 |
| TGCA | 0.007016 | -0.03902 | 6.89E-11 | AAGC | 0.001842 | 0.053415 | 7.83E-23 |
| TCTT | 0.006981 | 0.049647 | 8.22E-11 | TAAT | 0.006724 | 0.084251 | 1.06E-22 |
| GCTC | 0.004304 | 0.058381 | 1.89E-10 | GTTT | 0.004545 | 0.085808 | 6.17E-22 |
| ATTT | 0.00348 | 0.109838 | 2.98E-10 | TCAT | 0.004709 | -0.05711 | 1.43E-21 |
| GGGG | 0.004113 | -0.07355 | 4.87E-10 | CAGT | 0.004517 | -0.03452 | 2.53E-21 |
| AGTT | 0.003128 | 0.068023 | 6.22E-10 | TAAG | 0.005092 | -0.06785 | 1.59E-20 |
| CAAA | 0.010803 | 0.042564 | 8.86E-10 | TAAC | 0.003512 | -0.0546 | 3.56E-20 |
| CTGC | 0.003641 | -0.09002 | 1.42E-09 | TCTT | 0.007088 | 0.050914 | 5.44E-20 |
| GTCT | 0.002107 | 0.100542 | 1.55E-09 | GAGG | 0.003325 | 0.109886 | 1.14E-19 |
| CCTG | 0.017211 | -0.05636 | 3.04E-09 | GAAC | 0.002913 | 0.034857 | 3.11E-19 |
| TTAA | 0.002778 | -0.1095 | 3.07E-09 | CCTG | 0.017053 | -0.0619 | 9.62E-19 |
| TCCC | 0.004063 | 0.068853 | 3.17E-09 | GGGG | 0.004064 | -0.07463 | 2.41E-18 |
| CTTC | 0.004068 | -0.05587 | 4.73E-09 | GCCA | 0.008421 | 0.052791 | 2.96E-18 |
| GCTA | 0.004352 | 0.046988 | 7.40E-09 | AGTA | 0.002678 | -0.06262 | 3.24E-18 |
| GATG | 0.003436 | 0.037227 | 9.93E-09 | GCTC | 0.004302 | 0.057627 | 4.73E-18 |
| ACTT | 0.004481 | 0.082049 | 1.04E-08 | TTCA | 0.001842 | 0.092043 | 9.91E-18 |
| GAGG | 0.003415 | 0.102465 | 1.29E-08 | CCTA | 0.008099 | -0.05579 | 4.53E-17 |
| CGGA | 0.000827 | -0.06299 | 1.65E-08 | ACCA | 0.005184 | 0.045742 | 1.61E-16 |
| CTCA | 0.004679 | -0.06893 | 1.86E-08 | GTTG | 0.002004 | 0.048167 | 3.49E-16 |
| AACA | 0.003937 | 0.043716 | 1.97E-08 | ATTT | 0.003577 | 0.10191 | 4.17E-16 |
| ACGG | 0.000238 | 0.155745 | 1.98E-08 | ACCC | 0.003454 | 0.044998 | 4.64E-16 |
| GCCA | 0.008416 | 0.049805 | 2.12E-08 | TTAA | 0.00282 | -0.1068 | 1.09E-15 |
| GTGG | 0.001759 | 0.139229 | 2.16E-08 | GGGT | 0.002896 | -0.04417 | 1.17E-15 |
| TTTA | 0.003328 | -0.06557 | 2.20E-08 | AGTT | 0.003174 | 0.067667 | 3.12E-15 |
| GTCA | 0.001721 | 0.095137 | 2.22E-08 | CTGG | 0.004657 | -0.08893 | 4.21E-15 |
| ACCC | 0.003421 | 0.046605 | 3.35E-08 | CGCC | 0.001456 | -0.08931 | 7.99E-15 |
| CGCT | 0.001124 | -0.0574 | 6.98E-08 | AACA | 0.003995 | 0.04663 | 2.66E-14 |
| GGGT | 0.002923 | -0.04298 | 7.38E-08 | GATG | 0.00344 | 0.032942 | 3.87E-14 |
| ACCA | 0.005137 | 0.04509 | 1.03E-07 | CGGA | 0.000806 | -0.0637 | 5.09E-14 |
| CTGG | 0.004809 | -0.08289 | 1.12E-07 | ACTT | 0.004638 | 0.083125 | 8.57E-14 |
| AAGC | 0.001853 | 0.050421 | 1.57E-07 | TCCC | 0.004016 | 0.069821 | 3.16E-13 |
| ACAT | 0.004578 | -0.05274 | 1.74E-07 | GGAT | 0.004628 | -0.02722 | 3.29E-13 |
| GCGT | 0.000276 | 0.155924 | 1.99E-07 | ACAT | 0.004711 | -0.05348 | 6.61E-13 |
| ACTA | 0.002891 | 0.0639 | 2.22E-07 | TGCA | 0.007007 | -0.03813 | 7.66E-13 |
| CGGC | 0.001086 | -0.07524 | 7.97E-07 | TCGT | 0.000193 | 0.156392 | 1.08E-12 |
| TCAG | 0.004357 | -0.10279 | 8.23E-07 | ATCA | 0.001512 | 0.05215 | 1.37E-12 |
| TCGT | 0.000208 | 0.143857 | 5.09E-06 | ATAC | 0.001189 | -0.06568 | 5.73E-12 |
| GTTG | 0.002023 | 0.05936 | 7.18E-06 | TTTA | 0.003412 | -0.07164 | 5.88E-12 |
| GCCT | 0.008579 | 0.046415 | 7.93E-06 | GCCT | 0.008663 | 0.049223 | 1.72E-11 |
| TGGG | 0.006419 | -0.07555 | 9.37E-06 | GCTA | 0.004383 | 0.038158 | 4.07E-11 |
| TTCA | 0.001905 | 0.084751 | 1.80E-05 | ACTA | 0.002952 | 0.06089 | 4.39E-11 |
| CCTA | 0.008063 | -0.04868 | 2.00E-05 | ACTG | 0.004665 | -0.03903 | 9.42E-11 |
| TACA | 0.006307 | -0.03725 | 2.34E-05 | TGGG | 0.006343 | -0.07229 | 1.30E-10 |
| GCGC | 0.000294 | 0.139016 | 2.86E-05 | CGGC | 0.001056 | -0.07972 | 2.81E-10 |
| TCCT | 0.005438 | 0.034782 | 3.16E-05 | AACG | 0.000436 | 0.029141 | 6.28E-10 |
| AACG | 0.000436 | 0.029295 | 3.45E-05 | TCAG | 0.00439 | -0.10806 | 6.63E-10 |
| ACTG | 0.004572 | -0.03501 | 3.92E-05 | GCGC | 0.000276 | 0.157519 | 6.70E-10 |
| ATAC | 0.001189 | -0.05887 | 5.76E-05 | CGCT | 0.001102 | -0.05757 | 7.03E-10 |
| CCTC | 0.011048 | -0.03219 | 7.06E-05 | CCTC | 0.010903 | -0.03703 | 1.53E-09 |
| CAGA | 0.006985 | -0.02694 | 7.72E-05 | TACA | 0.006433 | -0.03331 | 2.67E-09 |
| ACGA | 0.000248 | 0.072182 | 0.00013592 | CAGA | 0.006911 | -0.02519 | 8.41E-09 |
| ATCA | 0.001523 | 0.052494 | 0.000185936 | TCCT | 0.005434 | 0.035648 | 7.21E-08 |
